# Allergic Disease with Selective IgA Deficiency. Comment on Cinicola et al. The Allergic Phenotype of Children and Adolescents with Selective IgA Deficiency: A Longitudinal Monocentric Study. *J. Clin. Med.* 2022, *11*, 5705

**DOI:** 10.3390/jcm12216703

**Published:** 2023-10-24

**Authors:** Öner Özdemir

**Affiliations:** Division of Allergy and Immunology, Department of Pediatrics, Research and Training Hospital of Sakarya University Medical Faculty, Adnan Menderes Cad., Sağlık Sok., No: 195, Adapazarı 54100, Türkiye; onerozdemir@sakarya.edu.tr; Tel.: +90-(264)-444-5400; Fax: +90-(264)-275-9192

I read the article titled ‘**The Allergic Phenotype of Children and Adolescents with Selective IgA Deficiency: A Longitudinal Monocentric Study**’ by Cinicola et al. with great interest. Nevertheless, there are a couple of questions raised in my mind about their study. And the clarification of these issues will help the reader to better understand and benefit from the study.

First, in the study [1], the authors said that 59/67 fulfilled the criteria for absolute SIgAD, whereas the remaining eight patients had partial SIgAD. Identifying some of the cases of both selective and partial deficiency (partial SIgAD) for above >7 mg/dL serum IgA levels is not easily understandable, especially in reference to guidelines [2,3]. It would be better termed ‘partial IgA deficiency’ not ‘partial selective’ IgA deficiency. Also, the terminology of absolute SIgAD may also be perplexing [1].

Categorizing selective IgA deficiency (SIgAD) as absolute (aSIgAD) or partial (pSIgAD) when the serum IgA level is <7 mg/dL or 2 SD below normal for a given age is not easily comprehensible. These definitions (aSIgAD and pSIgAD) mentioned are not universal and not accepted by everyone or by every guideline. For instance, the ESID (European Society for Immunodeficiencies) simply uses the term ‘definitive’ IgA deficiency instead of aSIgAD or ‘probable’ for pSIgAD [2]. The ESID does not even use the term SIgAD but rather IgA deficiency. While giving these definitions, I think it would be more accurate to define them according to generally accepted international guidelines, e.g., the ESID, not according to the inclusion criteria used in the research study [4].

Second, it is said in the article that patients showed more frequent transitory lymphopenia at diagnosis. When I examined the (IQR) of absolute lymphocyte numbers in Table 1, none of them were below 1.5 × 10^9^/L [5], and even the median was just below 2.0 × 10^9^/L. The (IQR) of absolute lymphocyte numbers was 1.9–2.8 × 10^9^/L. What was the cut-off number of lymphopenia in this study? Could the authors provide the minimum values for lymphopenia?

In hemograms, the lymphocyte count range varies based on age, and lymphopenia is defined according to age, namely, absolute lymphocyte count <3.0 × 10^9^/L in children younger than 2 years, <2.0 × 10^9^/L in children aged 2–5 years, and <1.5 × 10^9^/L in the 6–18-year age group [6,7]. Lymphopenia is not defined as <3000/mm^3^ in all children but particularly in infants (especially in infants of 1–12 months of age; however, this might extend up to 2 years of age). However, all the patients in this study [1] were above 4 years of age, and the minimum age was 7 years. For instance, in Table 1, the median (IQR) age of the patients was 9 (7–13.5) years and the median of their lymphocyte counts was 2.1 at the beginning of the study and 2.3 at the last follow-up [1]. In order to be able to say that a patient had lymphopenia in this study, their CBC should have had less than 1.500 lymphocytes/mm^3^.

Third, in Table 5 [1], there is a minor point and spelling error. Igg mg/dL should be IgG mg/dL.

In conclusion, I would like to thank the authors for this high-quality study and its results and their comments. In particular, this study shows us that SIgAD patients may present with allergic complaints other than recurrent infections. This is a study that will also pave the way for future work.
